# Management of pain in individuals with spinal cord injury: Guideline of the German-Speaking Medical Society for Spinal Cord Injury

**DOI:** 10.3205/000271

**Published:** 2019-06-17

**Authors:** Steffen Franz, Barbara Schulz, Haili Wang, Sabine Gottschalk, Florian Grüter, Jochen Friedrich, Jean-Jacques Glaesener, Fritjof Bock, Cordelia Schott, Rachel Müller, Kevin Schultes, Gunther Landmann, Hans Jürgen Gerner, Volker Dietz, Rolf-Detlef Treede, Norbert Weidner

**Affiliations:** 1 Spinal Cord Injury Center, Heidelberg University Hospital, Heidelberg, Germany; 2 BG Klinikum Bergmannstrost, Abteilung Medizinische Psychologie, Spezielle Traumatherapie (DeGPT), Hypnotherapie und Hypnose (DGH), Halle, Germany; 3 Zentralklinik Bad Berka GmbH, Querschnittgelähmten-Zentrum/Klinik für Paraplegiologie und Neuro-Urologie, Bad Berka, Germany; 4 Kliniken Beelitz GmbH, Neurologische Rehabilitationsklinik, Beelitz-Heilstätten, Germany; 5 Elbland Reha- und Präventions-GmbH, Großenhain, Germany; 6 BG Hospital Hamburg, Center for Rehabilitation, Hamburg, Germany; 7 Orthopädie am Grünen Turm, Ravensburg, Germany; 8 Orthopädische Privatpraxis Schott (OPS), Im Medizinischen Zentrum Essen, Germany; 9 Swiss Paraplegic Research, Nottwil, Switzerland; 10 Fördergemeinschaft der Querschnittgelähmten in Deutschland e.V., Lobbach, Germany; 11 Center for Pain Medicine, Swiss Paraplegic Centre, Nottwil, Switzerland; 12 Spinal Cord Injury Center, University Hospital Balgrist, Zurich, Switzerland; 13 Chair of Neurophysiology, Centre of Biomedicine and Medical Technology Mannheim, Heidelberg University, Mannheim, Germany

**Keywords:** neuropathic pain, nociceptive pain, central pain syndrome, clinical practice guideline, spinal cord injury, pain management, drug therapy, non-pharmacological pain therapy

## Abstract

**Introduction:** Pain is a prominent complication in spinal cord injury (SCI). It can either occur as a direct or as an indirect consequence of SCI and it often heavily influences the quality of life of affected individuals. In SCI, nociceptive and neuropathic pain can equally emerge at the same time above or below the level of injury. Thus, classification and grading of pain is frequently difficult. Effective treatment of SCI-related pain in general and of neuropathic pain in particular is challenging. Current treatment options are sparse and their evidence is considered to be limited. Considering these aspects, a clinical practice guideline was developed as basis for an optimized, comprehensive and standardized pain management in SCI-related pain.

**Methods:** The German-Speaking Medical Society for Spinal Cord Injury (Deutschsprachige Medizinische Gesellschaft für Paraplegiologie – DMGP) developed a clinical practice guideline that received consensus from seven further German-speaking medical societies and one patient organization. The evidence base from clinical trials and meta-analyses was summarized and subjected to a structured consensus-process in accordance with the regulations of the Association of Scientific Medical Societies in Germany (AWMF) and the methodological requirements of the “German instrument for methodological guideline appraisal”.

**Results:** This consensus-based guideline (S2k classification according to the AWMF guidance manual and rules) resulted in seven on-topic statements and 17 specific recommendations relevant to the classification, assessment and therapy of pain directly or indirectly caused by SCI. Recommended therapeutic approaches comprise pharmacological (e.g. nonsteroidal anti-inflammatory drugs or anticonvulsants) and non-pharmacological (e.g. physical activity or psychotherapeutic techniques) strategies for both nociceptive and neuropathic pain.

**Discussion:** Assessment of SCI-related pain is standardized and respective methods in terms of examination, classification and grading of pain are already in use and validated in German language. In contrast, valid, evidence-based and efficient therapeutic options are limited and ask for further clinical studies, ideally randomized controlled trials and meta-analyses.

## Zusammenfassung

**Einleitung:** Schmerzen sind eine höchst relevante Komplikation nach erlittener Querschnittlähmung. Sie können als eine direkte oder indirekte Folge auftreten und erheblichen Einfluss auf die Lebensqualität Betroffener haben. Nach einer Querschnittlähmung können nozizeptive und neuropathische Schmerzen gleichermaßen und zeitgleich sowohl oberhalb als auch unterhalb des Lähmungsniveaus auftreten. Deshalb erscheinen Klassifikation und Einordnung der Schmerzen häufig problematisch. Die Behandlung dieser Schmerzen ist generell schwierig und insbesondere für neuropathische Schmerzen herausfordernd. Dies ergibt sich nicht zuletzt aus den wenig vorhandenen und kaum evidenzbasierten Behandlungsmöglichkeiten. In Anbetracht dessen soll die Leitlinie als valide Basis für ein optimiertes, umfassendes und standardisiertes Schmerzmanagement dienen.

**Methoden:** Sieben deutschsprachige medizinische Fachgesellschaften mit thematischem Bezug zur Leitlinie und eine Fördergemeinschaft Betroffener entwickelten unter Federführung der Deutschsprachigen Medizinischen Gesellschaft für Paraplegiologie (DMGP) diese Leitlinie mittels einer strukturierten Konsensfindung nach den Maßgaben des Regelwerks der Arbeitsgemeinschaft der Wissenschaftlichen Medizinischen Fachgesellschaften (AWMF) sowie in Übereinstimmung mit dem deutschen Instrument zur methodischen Leitlinienbewertung.

**Ergebnisse:** Diese Leitlinie (S2k-Klassifikation gemäß dem Regelwerk der AWMF) ergab einen einstimmigen Konsens für sieben Kernaussagen und 17 spezifische Empfehlungen mit Bezug zur Klassifikation, Untersuchung/Beurteilung und Therapie von direkt oder indirekt infolge einer Querschnittlähmung auftretender Schmerzen. Empfohlene Therapien umfassen sowohl medikamentöse (z.B. nicht-steroidale Antirheumatika und Antikonvulsiva), als auch nicht-medikamentöse (z.B. körperliche Aktivität und psychotherapeutische Maßnahmen) Ansätze und betreffen nozizeptive wie auch neuropathische Schmerzen.

**Diskussion:** Es existieren diverse standardisierte und verlässliche Methoden zur Beurteilung und Untersuchung von Schmerzen nach Querschnittlähmung, die auch häufig in deutscher Sprache validiert sind. Demgegenüber sind evidenzbasierte und effektive schmerztherapeutische Möglichkeiten stark begrenzt und bedürfen weiterer wissenschaftlicher Auseinandersetzung, die sich im Idealfall aus randomisierten kontrollierten Studien und Metaanalysen ergibt.

## Introduction

Acute and chronic pain are crucial complications in the course of spinal cord injury (SCI), entailing serious impacts not only on the primary rehabilitation, but also on the individuals’ quality of life in later phases of SCI [1], [2], [3]. The vast majority of individuals with SCI experiences pain of any manifestation at some time after injury [4], [5]. Most frequently, pain occurs within the early phase of disease but also commonly emerges in later stages, for instance, as consequence of complications in direct or indirect relation to SCI [4], [6]. Hence, numerous varieties of pain presentation are common in SCI. These presentations can generally be assigned to different pain types, some of which are very common, like nociceptive and/or neuropathic pain. Such pain types can be present at and/or below the neurological level of injury (NLI), but also in body regions that are not affected by SCI.

Nociceptive pain is defined as pain that is caused by either an irritation or an injury of body tissue, without an associated impairment of somatosensory structures [7]. This type of pain can be addressed by means of a causal treatment but could also evolve into a chronic condition. Three subtypes are common in SCI: 1) musculoskeletal, 2) visceral, and 3) other nociceptive pain. While musculoskeletal pain in SCI is mostly due to mechanical damage with ensuing injury of bones (fractures), muscles, ligaments and joints [8], visceral pain is frequently resulting from constipation. Other nociceptive pain could be caused by pressure sores.

**Figure 1:**
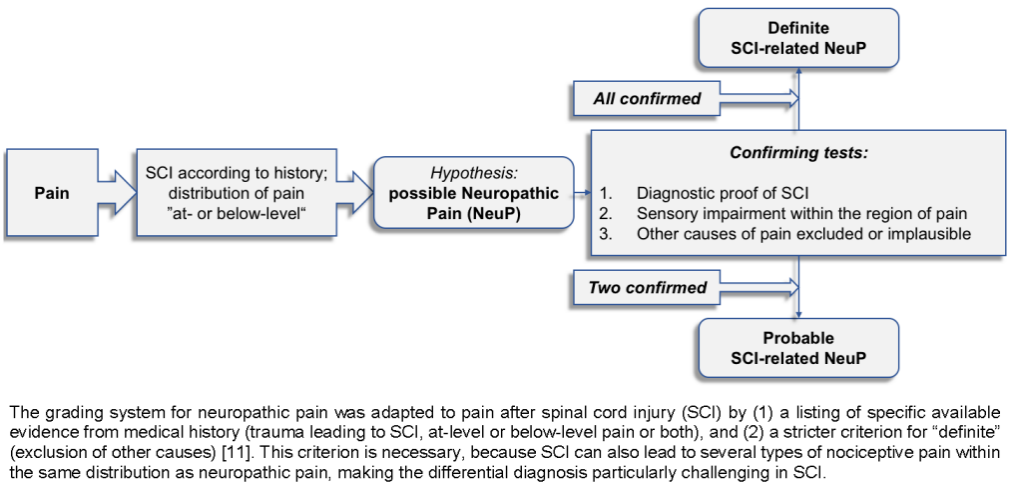
Adapted grading system for neuropathic pain after spinal cord injury (SCI) according to the International Association for the Study of Pain (IASP) [12]

According to the International Association for the Study of Pain, neuropathic pain is defined as “pain arising as a direct consequence of a lesion or disease affecting the somatosensory system” [9]. ‘Definite neuropathic pain’ can be diagnosed if medical history suggests a disease that could potentially lead to a lesion of the somatosensory system, *and* if the pain is distributed in a neuroanatomically plausible pattern (e.g. according to a dermatome or a cutaneous innervation), *and* if the former, as well as the latter condition is confirmed by clinical or instrument-based (e.g. MRI) examinations [10]. This algorithm has recently been adapted for application in SCI (Figure 1 (Fig. 1)) [11], [12]. Neuropathic pain in general is further subdivided into central and peripheral neuropathic pain. While the latter is due to a lesion of peripheral neural structures (e.g. peripheral nerves or nerve roots), the former is related to an impairment of the central nervous system, like certain structures within the spinal cord (e.g. sensory neurons in the grey matter or afferent white matter tracts) [13]. Peripheral neuropathic pain frequently presents as, but is not limited to, evoked pain (e.g. tactile stimuli), whereas central neuropathic pain is characterized by spontaneous and continuous pain presentation [14], [15]. At-level neuropathic pain in SCI is localized at and/or within three levels below the NLI and can be caused by both lesions of the peripheral nervous system (e.g. nerve roots) and lesions of the spinal cord itself. In contrast, neuropathic pain which is localized more than three segments below the NLI is commonly considered as central and defined as below-level neuropathic pain [16]. Notably, pain in SCI can also be triggered by nociceptive stimuli below the NLI, which frequently cannot be perceived by the patient (e.g. bladder infection). Thus, classification and grading of pain can be quite challenging, particularly if causes of pain are located below the NLI [17].

Except for some evidence reporting that cervical lesions might be related to a higher probability to develop central neuropathic pain, no further predisposing factors are known, yet. Neither the severity of SCI, nor the NLI are proven to have influence on the emergence of distinct pain types or the intensity of perceived pain [18].

Concerning the efficient treatment of SCI-related pain, there are two meta-analyses on pharmacotherapy in general and one on gabapentinoids in particular, one on a surgical intervention (DREZ lesion) and one on physical or behavioral treatment [19], [20], [21], [22], [23]. Overall, however, evidence is still sparse and therapeutic options are limited, especially with regard to neuropathic pain. According to current understandings, optimized management of pain in SCI is dependent on a well-matched personalized approach, which involves both pharmacological and non-pharmacological means [3].

All these evident specifics and challenges emphasize the need of a systematic and structured approach for pain management in SCI, such as clinical practice guidelines. Accordingly, the primary goal of this clinical practice guideline is to establish and standardize a broadly approved and recognized concept to address the frequent challenges regarding the management of SCI-related pain syndromes in German-speaking countries. This in turn will likely improve health care in affected SCI individuals and represents a basis to develop more effective therapeutic options in the future.

This guideline addresses physicians and therapists of all participating medical societies and specialist disciplines (e.g. neurologists, physiatrists, pain specialists, orthopedists, psychotherapists). It should serve as a source of information for all further professional fields that are involved in the treatment of adult individuals with acute and chronic spinal cord injury in out- and inpatient setting.

## Methods

This consensus-based clinical practice guideline was developed by the German-Speaking Medical Society for Spinal Cord Injury (Deutschsprachige Medizinische Gesellschaft für Paraplegiologie, DMGP) in accordance with the applicable regulations of the Association of Scientific Medical Societies in Germany (Arbeitsgemeinschaft der Wissenschaftlichen Medizinischen Fachgesellschaften e.V., AWMF) and the methodological requirements of the German instrument for methodological guideline appraisal (DELBI) [24], [25]. According to the three-stage concept of the AWMF it is a S2k guideline. The AWMF is representing Germany in the Council for International Organizations of Medical Sciences (CIOMS; https://cioms.ch).

### Methodological accuracy

An evidence base derived from Pubmed^®^, Medline^®^, and the Cochrane Library, as well as statements from preceding international guidelines were subjected to a structured consensus-building process [26], [27], [28]. Statements with regard to taxonomy, classification, and diagnostic procedures are based on widely accepted algorithms and tools that had already been evaluated, rated, and endorsed by international societies like the International Spinal Cord Society (ISCoS), the American Spinal Injury Association (ASIA), or the International Association for the Study of Pain (IASP) [10], [11], [29], [30], [31]. Accordingly, non-controversial statements were evaluated within the regular review process of the guideline content (background text) without a formal voting procedure (1^s^^t^ through 4^th^ revision within the guideline panel; see Table 1 (Tab. 1)). All representatives of the guideline panel had the opportunity to comment on each statement and the full content of the background text by use of a form throughout the review process. 

In contrast, relevant pharmacological and non-pharmacological therapeutic approaches are frequently based on sparse or even conflicting evidence. In particular, pharmacological interventions are tightly regulated by authorities varying from country to country. Thus, potential therapeutic options were rated by means of a structured voting procedure (3^rd^ and 4^th^ revision within the guideline panel). The evaluation of existing evidence was oriented towards the GRADE process (Grading of Recommendations Assessment, Development and Evaluation) [32], [33]. Correspondingly, the pre-defined specifications to appraise the strength of recommendations are listed in Table 2 (Tab. 2). Considering the limited standardization of literature review, the quality of identified evidence was not explicitly rated and expressed with descriptions like “high”, “moderate”, or “(very) low” as it is exemplarily specified according to GRADE. Nevertheless, the analysis of existing evidence influenced the wording of stated recommendations for pharmacological and non-pharmacological therapies, which were graded on a three-level scale according to Table 3 (Tab. 3). The strength of consensus was finally determined by the approval rating for each recommendation (Table 4 (Tab. 4)). 

After completion of the consensus within the panel, the guideline was submitted to the AWMF for a final external review (methodological monitoring), to the board of the DMGP guideline commission, as well as to all authorized bodies of the involved associations and societies for a concluding content review.

### Constitution and structure of the guideline panel

Commissioned by the DMGP, the corresponding author convened a representative committee and coordinated the structured consensus building of the guideline project. The guideline panel was subdivided into a steering committee (Table 5 (Tab. 5)), constituted of experts from different disciplines – neurologists, psychologists, pain specialists, a physiatrist, and a physiologist – who are involved in the diagnosis and treatment of SCI, and a group of appointed representatives from seven German-speaking scientific medical societies and one patient organization (Table 6 (Tab. 6)).

### Process of structured consensus building

During a project kick-off meeting the steering committee decided on outline and main content of the planned guideline. Subsequently, each member of the steering committee was assigned to a work group charged with a specific topic of the guideline and commissioned to prepare a topic-related draft. Thereafter, the drafts were combined to a first preliminary version of the guideline, which again went through a review process, firstly within the steering committee. Within this process, all committee members were required to screen existing evidence/literature, available international guidelines and relevant recommendations of international societies. In addition, committee members contributed their own reasoned expert opinion. 

The structured process of consensus building within the whole guideline panel was initiated after the conclusion of the steering committees’ review process. Representatives of each aforementioned society/association and members of the steering committee were asked to comment and edit the guideline as appropriate (1^st^ and 2^nd^ revision within the guideline panel). In each of the two rounds, the steering committee prepared a revised version of the guideline for re-submission to the group. Subsequently, the coordinator of the consensus-building process analyzed the preliminary guideline in terms of core statements and grading of recommendations. The core statements and recommendations were then summarized to be voted upon (3^rd^ and 4^th^ revision within the guideline panel). All members of the guideline panel were required to judge about every single item. In case of dissent, each member had to either justify the decision or to provide alternative suggestions. Whenever possible, a dissent needed to be supported by relevant published evidence. Suggestions were submitted to the guideline panel along with a commented revised draft of the guideline, which was previously prepared by the steering committee. All members were asked to decide about the revised proposals and to comment on the suggestions of the steering committee if necessary. This procedure was iterated until a unanimous consent was achieved. Throughout the process of guideline development, all group members were called upon to check for recently published or by then missed literature and evidence, respectively. Furthermore, throughout the guideline development emerging expert statements in the context of pain treatment could be incorporated if relevant. A summary of the structured consensus building is given in Table 1 (Tab. 1). In a concluding survey among all members of the panel, the guideline was finally consented and approved by the participating societies.

## Results

The structured consensus process led to a clinical practice guideline of S2k classification according to the AWMF guidance manual and rules [24] and resulted in the following statements and recommendations arranged according to different groups of themes:

### Classification of SCI-related pain

- **Statement 1.1**
  The classification of SCI-related pain ought to be done according to the international spinal cord injury pain (ISCIP) classification (Table 7 (Tab. 7)) [30], [31].

### Diagnosis of SCI-related pain

#### General considerations regarding diagnostic routines

- **Statement 2.1**
  Diagnosis of SCI-related pain ought to be done in a structured and standardized manner. Therefore, it should be based on the recently published and widely accepted International Spinal Cord Injury Pain Datasets (ISCIPD) [34], [35].
- **Statement 2.2**
  In case psychological factors influence pain perception, this ought to be considered as “chronic pain disorder with somatic and psychological factors” according to the international statistical classification of diseases and related health problems (ICD-10), published by the World Health Organization (WHO) [36].
- **Statement 2.3**
  Yet, the ISCIPD is not translated and linguistically validated in German. Thus, the German pain questionnaire (Deutscher Schmerzfragebogen, DSF) may serve as a basis with respect to the patient medical history and screening for psychological comorbidities [37].

#### Basic notes on the clinical examination as part of diagnostic routines

- **Statement 3.1**

1.
  1.
    1. Nociceptive pain ought to be examined by means of an accurate past medical history record and a clinical examination of all body regions that are potentially exposed to substantial physical exertion in the wake of SCI.
  2.
    1. Visceral pain is most commonly due to complications regarding neurogenic bladder and bowel dysfunction.

- **Statement 3.2**
  SCI-related neuropathic pain should be clinically evaluated as follows (Figure 1 (Fig. 1)) [38]:
  - Onset of pain within the first year of injury?
  - No competing causes of pain (e.g. scars, skin wounds, ulcers)?
  - Presentation of pain has no dependency on movement/manipulation of the painful area?
  - One or more of the following pain qualities: “hot/burning”, “tingling”, “electrical“, “constricting/ squeezing” or “freezing”?
  - Is the painful area located in a body region of abnormal sensory function (hypesthesia, allodynia, hyperalgesia)?
- **Statement 3.3**
  Supplementary to the clinical examination, various questionnaires and scales are available to evaluate pain [39], [40], [41], [42].

#### Basic notes on the medical diagnostics

- **Statement 4.1**
  Depending on the cause, assessment of nociceptive pain can be supported by several diagnostic measures. Most commonly, imaging (sonography, X-ray diagnostics, computed tomography [CT], magnetic resonance imaging [MRI]) is the preferred approach to further evaluate the causes of nociceptive pain with respect to its underlying structural changes.
- **Statement 4.2**
  For affirming the diagnosis of SCI-related neuropathic pain, imaging techniques are preferably used to detect a spinal cord lesion (CT, MRI). Furthermore, neurophysiological measures can help to evaluate the integrity of relevant spinal tracts. Well established techniques include somatosensory evoked potentials (SSEP) for examining lemniscal functions, contact heat evoked potentials (CHEPS) and laser evoked potentials (LEP) for testing nociceptive and thermoreceptive tracts and motor evoked potentials (MEP) for assessing the corticospinal tract [43], [44], [45], [46], [47], [48], [49], [50].
- **Statement 4.3**
  The Quantitative Sensory Testing (QST) may be considered in case of uncertainty concerning the interpretation of symptoms with respect to neuropathic pain [51], [52], [53], [54].

### Prediction and prevention of pain in SCI

- **Statement 5.1**
  Conclusive evidence to prevent and predict pain after SCI is missing [55].
- **Statement 5.2**
  Potential risk factors for pain chronification, such as age or an early onset of pain, are being subject to ongoing discussions.
  However, it is generally accepted that the avoidance of secondary complications after SCI is of utmost importance to diminish the risk of nociceptive pain development and its chronification [56], [57].
- **Statement 5.3**
  Early presentation of allodynia in certain skin areas might be a predictor of developing neuropathic pain [54].

### Considerations regarding treatment of SCI-related pain

#### Expectations on treatment

- **Statement 6.1**
  Therapeutic goal setting ought to be reasonable and realistic. Complete pain relief is unlikely to occur.

#### Current level of evidence

- **Statement 6.2**
  As a basis for designated treatment recommendations, five meta-analyses exist, especially concerning pharmacological therapeutic approaches.
  However, existing evidence on treatment of SCI-related pain is sparse, and further research in terms of randomized controlled trials (RCT) is required [19], [20], [21], [22], [23].

#### General considerations

- **Statement 6.3**
  A timely and sufficient treatment of pain, irrespective of the pain type and based on current pathophysiological insights, is of utmost importance [58].
- **Statement 6.4**
  The primary therapeutic objective is to treat underlying injuries, causes and triggers.

#### Specific considerations in relation to nociceptive pain

- **Statement 6.5**
  The WHO’s analgesic ladder including adjuvants serves as basic guidance for treatment of nociceptive pain [59].
- **Statement 6.6**
  Indications and side effects of analgesics have to be evaluated in respect to their particular relevance in SCI (Table 8 (Tab. 8)).
- **Statement 6.7**
  Based on broad clinical expert experience, nonsteroidal anti-inflammatory drugs (NSAR) are considered to be effective in acute nociceptive pain.
- **Statement 6.8**
  The adequate supply of medical aids plays a central role with respect to the treatment of nociceptive pain [60].

#### Specific considerations in relation to neuropathic pain

- **Statement 6.9**
  Treatment of neuropathic pain is primarily a symptomatic therapy, unless underlying causes can be addressed [61].
- **Statement 6.10**
  Adjuvants, which are in accordance with the WHO’s analgesic ladder are considered to be equivalent to the drugs that also have a therapeutic effect on neuropathic pain.
- **Statement 6.11**
  There is a lack of evidence regarding efficacy of NSAR in neuropathic pain relief.

### Pharmacological options for treatment of SCI-related pain

#### Anticonvulsants

**RECOMMENDATION 1.1: PREGABALIN**

INDICATION FOR THE TREATMENT OF NOCICEPTIVE PAIN

Degree of recommendation and related specifics: **n/a**

Strength of consent: **n/a**

*INDICATION FOR THE TREATMENT OF NEUROPATHIC PAIN*

Degree of recommendation and related specifics: ↑↑

Strength of consent: **strong**

DOSAGE AND ADMINISTRATION

Approved daily maximum dose:

**600 mg in 2 or 3 single doses**

Dose increase:

**weekly, starting with 150 mg p.d. (per day)**

BASIC INFORMATION AND BODY OF EVIDENCE

Pregabalin is recommended to be applied as first-line therapy. This is based on one meta-analysis and four RCT, showing a positive effect on pain relief, occasionally in lower doses, but most commonly in doses of =300 mg [23], [62], [63], [64], [65].

**RECOMMENDATION 1.2: GABAPENTIN**

INDICATION FOR THE TREATMENT OF NOCICEPTIVE PAIN

Degree of recommendation and related specifics: **n/a**

Strength of consent: **n/a**

*INDICATION FOR THE TREATMENT OF NEUROPATHIC PAIN*

Degree of recommendation and related specifics: ↑↑

Strength of consent: **strong**

DOSAGE AND ADMINISTRATION

Approved daily maximum dose:

**3600 mg in 3 single doses**

1^st^ day: **100 mg t.i.d. (three times a day)**

2^nd^ day: **200 mg t.i.d.**

3^rd^ day: **300 mg t.i.d.**

From then on: **increase by 300 mg p.d., every other day**

BASIC INFORMATION AND BODY OF EVIDENCE

Gabapentin is recommended to be applied as first-line therapy, if and when undesirable side effects occur or efficiency is unsatisfactory with pregabalin. Use of gabapentin is off-label for central neuropathic pain (below-level), however approved for peripheral neuropathic pain (at-level) in Germany. Tolerability of its application has to be monitored continuously. After one week of administration, 1800 mg p.d. should not be exceeded; 2400 mg p.d. should be reached earliest after two weeks; and 3600 mg should not be given before three weeks after the first administration. This is based on one meta-analysis and two RCT [23], [66], [67]. Another RCT could not demonstrate a significant therapeutic effect of gabapentin [68], while 3 small non-randomized studies also suggest a beneficial effect of gabapentin on SCI-related neuropathic pain.

**RECOMMENDATION 1.3: LAMOTRIGINE**

INDICATION FOR THE TREATMENT OF NOCICEPTIVE PAIN

Degree of recommendation and related specifics: **n/a**

Strength of consent: **n/a**

*INDICATION FOR THE TREATMENT OF NEUROPATHIC PAIN*

Degree of recommendation and related specifics:

↓**/in complete lesions**

↔**/only in incomplete lesions**

Strength of consent: **strong**

DOSAGE AND ADMINISTRATION

Trialed daily maximum dose:

**400 mg in 1 or 2 single doses**

1^st^ two weeks: **25 mg q.d. (once a day)**

2^nd^ two weeks:** 50 mg q.d.**

2^nd^ month: **100 mg q.d. or 50 mg twice a day**

Thereafter: in weekly intervals increase by 100 mg

BASIC INFORMATION AND BODY OF EVIDENCE

Lamotrigine is not suggested to be applied in complete SCI and may be considered as reserve drug (third-line) in incomplete SCI. In one RCT (n=22) a subgroup analysis implied an effect on perception of pain intensity in incomplete SCI (n=12), with however limited statistical certainty [69], [70]. The known profile of possible side effects (Stevens-Johnson syndrome, dizziness, somnolence, etc.) has to be considered.

**RECOMMENDATION 1.4: OTHER ANTICONVULSANTS**

INDICATION FOR THE TREATMENT OF NOCICEPTIVE PAIN

Degree of recommendation and related specifics: **n/a**

Strength of consent: **n/a**

*INDICATION FOR THE TREATMENT OF NEUROPATHIC PAIN*

Degree of recommendation and related specifics: ↓↓

Strength of consent: **strong**

BASIC INFORMATION AND BODY OF EVIDENCE

According to the literature available, neither sodium valproate [71], [72] nor levetiracetam [73], [74] are recommended to be used in SCI-related neuropathic pain.

#### Antidepressants

**RECOMMENDATION 2.1: DULOXETINE**

INDICATION FOR THE TREATMENT OF NOCICEPTIVE PAIN

Degree of recommendation and related specifics: **n/a**

Strength of consent: **n/a**

*INDICATION FOR THE TREATMENT OF NEUROPATHIC PAIN*

Degree of recommendation and related specifics:

↑**/in case of additional depression and/or at-level neuropathic pain**

Strength of consent: **strong**

DOSAGE AND ADMINISTRATION

**60 mg or 120 mg q.d.**

BASIC INFORMATION AND BODY OF EVIDENCE

One RCT suggests a possible beneficial effect in SCI-related neuropathic pain as measured by VAS (p=0,05) [75]. Some evidence additionally proposes an effect on peripheral neuropathic pain in other underlying diseases [76]. Thus, duloxetine is suggested as alternative therapeutic option in case of additionally diagnosed depression and/or at-level neuropathic pain, and if patient-related conditions or other reasons discourage the use of anticonvulsants (e.g. emerging side effects). Duloxetine should be preferred over the tricyclic amitriptyline due to a more favorable profile of side effects [77], [78].

**RECOMMENDATION 2.2: AMITRIPTYLINE**

INDICATION FOR THE TREATMENT OF NOCICEPTIVE PAIN

Degree of recommendation and related specifics: **n/a**

Strength of consent: **n/a**

*INDICATION FOR THE TREATMENT OF NEUROPATHIC PAIN*

Degree of recommendation and related specifics:

↑**/in case of additional depression**

Strength of consent: **strong**

DOSAGE AND ADMINISTRATION

**150 mg q.d.**

BASIC INFORMATION AND BODY OF EVIDENCE

In one meta-analysis, amitriptyline is questioned as appropriate therapeutic agent in SCI-related neuropathic pain [22]. Two RCTs that investigated the efficiency of amitriptyline in SCI neuropathic pain yielded conflicting evidence [68], [79]. Accordingly, we suggest amitriptyline only if anticonvulsants and duloxetine do not show the desired effect and a diagnosis of depression is coincident. Notably, the authors emphasize the frequent occurrence of side effects of amitriptyline [22].

**RECOMMENDATION 2.3: VENLAFAXINE**

INDICATION FOR THE TREATMENT OF NOCICEPTIVE PAIN

Degree of recommendation and related specifics:

↔**/in case of additional depression**

Strength of consent: **strong**

*INDICATION FOR THE TREATMENT OF NEUROPATHIC PAIN*

Degree of recommendation and related specifics:

↓↓**/in case of below-level neuopathic pain**

↔**/in case of at-level neuropathic pain**

Strength of consent: **strong**

DOSAGE AND ADMINISTRATION

Trialed daily maximum dose: **300 mg in 1 single dose**

Initially: **37.5 mg q.d.**

From then on flexible increase depending on efficacy:

1^st^ week: **max. up to 75 mg q.d.**

2^nd^ week: **max. up to 150 mg q.d.**

until week 6: **max. up to 225 mg q.d.**

weeks 8 to 10: **max. up to 300 mg q.d**

BASIC INFORMATION AND BODY OF EVIDENCE

One RCT (n=123) tested the efficacy of venlafaxine on concomitant depression in individuals with SCI (primary endpoint) [80], along with its influence on both nociceptive and neuropathic pain as secondary endpoints. The minimal effective dose resulted in 150 mg per day. The authors discuss an efficacy of venlafaxine exclusively for nociceptive pain alone and in case of a mixed nociceptive and neuropathic pain syndrome, as assumed in case of an inconclusive result in SCIPI. Venlafaxine may be considered in cases of nociceptive and at-level neuropathic pain with concomitant depression. Venlafaxine is not recommended to be used in below-level neuropathic pain alone.

#### Opioids

- **Statement 7**
  Evidence concerning the administration of opioids for mild, moderate or severe pain in SCI is generally sparse and heterogeneous. Based on that and reflecting the relevant profile of potential adverse drug reactions, especially in SCI, opioids are only recommended to be applied as last resort [81], [82], [83], [84], [85].

**RECOMMENDATION 3.1: TRAMADOL**

INDICATION FOR THE TREATMENT OF NOCICEPTIVE PAIN

Degree of recommendation and related specifics:

↔**/necessity of risk-benefit analysis**

Strength of consent: **strong**

*INDICATION FOR THE TREATMENT OF NEUROPATHIC PAIN*

Degree of recommendation and related specifics:

↔**/necessity of risk-benefit analysis**

Strength of consent: **strong**

DOSAGE AND ADMINISTRATION

Trialed daily maximum dose: **100 mg q.i.d.**

Initially: **50 mg q.d.**

From then on: **flexible increase depending on efficacy**

BASIC INFORMATION AND BODY OF EVIDENCE

One RCT (n=35) demonstrated a positive effect on the perception of pain intensity in neuropathic pain after SCI, however lacked precision regarding its statistical conclusiveness [86]. Remarkably, 47.8% of participants (11 out of 23) within the verum group terminated the application of tramadol, which was mostly due to adverse events. In a recent meta-analysis investigating the effects of tramadol in neuropathic pain relief (including SCI-related neuropathic pain), the authors come to a precarious conclusion, given the low quality of existing studies [87].

**RECOMMENDATION 3.2: OXYCODONE**

INDICATION FOR THE TREATMENT OF NOCICEPTIVE PAIN

Degree of recommendation and related specifics:

↔**/necessity of risk-benefit analysis**

Strength of consent: **strong**

*INDICATION FOR THE TREATMENT OF NEUROPATHIC PAIN*

Degree of recommendation and related specifics:

↔**/as add-on to anticonvulsants**

↓**/as monotherapy**

Strength of consent: **strong**

BASIC INFORMATION AND BODY OF EVIDENCE

One observational study (n=54) reports amelioration of neuropathic pain intensity within a 3 months period of administration after concomitant application of anticonvulsants and oxycodone [88]. Based on the poor evidence and the less favorable profile of potential side effects, oxycodone may be considered in neuropathic pain treatment only as add-on therapy to anticonvulsants, weighing the risk-benefit ratio. It may be considered as last resort for SCI-related nociceptive pain. For lack of evidence, oxycodone is not suggested as monotherapy for SCI-related neuropathic pain.

#### Further pharmacological options for treatment of SCI-related pain

##### Spasmolytic drugs

**RECOMMENDATION 4.1: BOTULINUM TOXIN**

INDICATION FOR THE TREATMENT OF NOCICEPTIVE PAIN

Degree of recommendation and related specifics:

↔**/intramuscular injection**

Strength of consent: **strong**

*INDICATION FOR THE TREATMENT OF NEUROPATHIC PAIN*

Degree of recommendation and related specifics:

↓**/intramuscular injection**

↔**/in case of below-level neuropathic pain (subcutaneously)**

Strength of consent: **strong**

BASIC INFORMATION AND BODY OF EVIDENCE

A case series of 28 patients reported a therapeutic effect of botulinum toxin A (BOTOX^®^/Allergan) on both focal spasticity and pain [89]. The standard doses ranged between 10 and 118 units per muscle. The exact pain type was not specified in this study, though. The focus of botulinum toxin injections was on muscles remarkably affected by clinical signs of spasticity, suggesting that musculoskeletal pain was primarily addressed.

Referring to their own preliminary single-case study, a workgroup from Korea conducted a subsequent RCT with 40 included patients, discussing a positive therapeutic effect of subcutaneously injected botulinum toxin within the area of below-level neuropathic pain (200 units apportioned to 40 injection sites in one single area of pain, maximally representing 20% of the body surface area) [90], [91]. However, in the same study this effect could not be demonstrated for at-level neuropathic pain (n=9). More importantly, the authors already critically discuss the short period of observation (8 weeks post-injection) and an indeed significant, however relatively low efficacy of botulinum toxin.

Accordingly, intramuscularly injected botulinum toxin A may be considered for therapy of nociceptive pain, if associated with spasticity. Pending further evidence, subcutaneous injection of botulinum toxin may not yet be considered as a therapeutic option for neuropathic pain. A principle recommendation against the application of botulinum toxin A in SCI-related pain is not being pronounced, considering the conceivable interactions between spasticity, nociceptive pain, and neuropathic pain [92], [93].

**RECOMMENDATION 4.2: BACLOFEN**

INDICATION FOR THE TREATMENT OF NOCICEPTIVE PAIN

Degree of recommendation and related specifics:

↑**/when associated with spasticity**

Strength of consent: **strong**

*INDICATION FOR THE TREATMENT OF NEUROPATHIC PAIN*

Degree of recommendation and related specifics: ↓

Strength of consent: **strong**

BASIC INFORMATION AND BODY OF EVIDENCE

Three case series and one single RCT with only a small sample size describe a therapeutic effect of intrathecally administered baclofen on both spasticity and SCI-related nociceptive pain. Concerning the efficacy of baclofen on neuropathic pain, existing evidence is still conflicting [94], [95], [96], [97], [98], [99].

To date, orally administered baclofen has not been systematically evaluated in respect to SCI-related pain. In summary, we suggest an oral application of baclofen only in cases of nociceptive pain associated with spasticity.

##### Topical medication

**RECOMMENDATION 5: LIDOCAINE AND CAPSAICIN**

INDICATION FOR THE TREATMENT OF NOCICEPTIVE PAIN

Degree of recommendation and related specifics: **n/a**

Strength of consent: **n/a**

*INDICATION FOR THE TREATMENT OF NEUROPATHIC PAIN*

Degree of recommendation and related specifics: ↔**/in case of at-level neuropathic pain**

Strength of consent: **strong**

BASIC INFORMATION AND BODY OF EVIDENCE

To date, no studies exist that investigated the efficacy of lidocaine-medicated or capsaicin-medicated plaster and capsaicin cream in SCI-related neuropathic pain. In the light of an existing clinical practice guideline for treatment of chronic neuropathic pain [100], a therapeutic attempt may be considered if localized at-level neuropathic pain is resistant to other therapies or in case of intolerance towards other therapeutic options.

**RECOMMENDATION 6: AGENTS FOR INVASIVE USE (INTRAVENOUSLY/INTRATHECALLY)**

INDICATION FOR THE TREATMENT OF NOCICEPTIVE PAIN

Degree of recommendation and related specifics: ↓

Strength of consent: **strong**

*INDICATION FOR THE TREATMENT OF NEUROPATHIC PAIN*

Degree of recommendation and related specifics: ↓

Strength of consent: **strong**

BASIC INFORMATION AND BODY OF EVIDENCE

Evidence for the use of intravenously or intrathecally administered agents is generally scarce. Opioids (morphine and alfentanil), lidocaine, ketamine and clonidine have been tested [101], [102], [103], [104], [105], [106]. All of these studies however lack long-term investigations. Further shortcomings comprise considerable methodological flaws and limitations, like small sample sizes and conflicting results in various studies. Thus, we do not suggest these agents for the use in both nociceptive pain and neuropathic pain, especially when reflecting the risk-benefit ratio of such invasive approaches.

**RECOMMENDATION 7: CANNABINOIDS**

INDICATION FOR THE TREATMENT OF NOCICEPTIVE PAIN

Degree of recommendation and related specifics: ↓

Strength of consent: **strong**

*INDICATION FOR THE TREATMENT OF NEUROPATHIC PAIN*

Degree of recommendation and related specifics: ↓

Strength of consent: **strong**

BASIC INFORMATION AND BODY OF EVIDENCE

Efficacy of cannabinoids in pain related to SCI has been evaluated in one meta-analysis which analyzed two studies [22]. One study investigated the effect of tetrahydrocannabinol on spasticity related to chronic pain with inconclusive results [107]. The other study focused on the impact of dronabinol on neuropathic pain yielding negative findings with limited validity regarding its precision in terms of statistical conclusiveness (n=7) [108].

Nevertheless, experts and affected patients repeatedly describe positive effects of cannabinoids as compassionate use for musculoskeletal nociceptive pain in the wake of deteriorating spasticity. Among affected individuals, neuropathic pain is occasionally reported to respond to the administration of cannabinoids as well. Based on insufficient evidence combined with unfavorable side effects (e.g. constipation, drowsiness, xerostomia), the administration of cannabinoids cannot be suggested for the therapy of SCI-related pain.

### Non-pharmacological measures for treatment of SCI-related pain

**RECOMMENDATION 8: PHYSICAL ACTIVITY, EXERCISE AND PHYSIOTHERAPEUTIC MEASURES**

INDICATION FOR THE TREATMENT OF NOCICEPTIVE PAIN

Degree of recommendation and related specifics: ↑↑

Strength of consent: **strong**

*INDICATION FOR THE TREATMENT OF NEUROPATHIC PAIN*

Degree of recommendation and related specifics: ↑

Strength of consent: **strong**

BASIC INFORMATION AND BODY OF EVIDENCE

A recent meta-analysis suggests that physical activity has a mild to moderate beneficial effect on chronic musculoskeletal pain related to SCI and emphasizes its favorable benefit-risk profile [109]. Exemplarily, mild physical activity (stretching and suchlike) performed two times a week for the period of three months could positively influence nociceptive pain after SCI [110]. As another example, SCI-related shoulder pain could likewise be ameliorated by means of a specific therapeutic concept, comprising an hour-long instructional course about a specific personalized training or an eight-week tailored training program for implementation at home, including daily stretching and strengthening exercises by use of elastic resistance bands every other day [111], [112], [113]. From further cross-sectional studies it can also be assumed that physiotherapeutic interventions could in general have a positive influence on chronic pain [114], [115].

Even though well-designed RCT on this topic are still lacking [116], [117], [118], [119], physical activity and/or physiotherapeutic measures are recommended to treat SCI-related nociceptive pain. Based on the assumed positive impact of physical activity and physiotherapy on chronic pain in general, respective measures are suggested for non-pharmacological neuropathic pain treatment.

**RECOMMENDATION 9: PSYCHOTHERAPEUTIC TECHNIQUES**

INDICATION FOR THE TREATMENT OF NOCICEPTIVE PAIN

Degree of recommendation and related specifics: ↔

Strength of consent: **strong**

*INDICATION FOR THE TREATMENT OF NEUROPATHIC PAIN*

Degree of recommendation and related specifics: ↔

Strength of consent: **strong**

BASIC INFORMATION AND BODY OF EVIDENCE

Psychotherapeutic interventions have not been extensively evaluated with respect to their efficacy in SCI-related pain [116], [120]. In reference to the concept of a multimodal therapeutic approach, both psychotherapeutic and psychoeducational strategies may however serve as a basis to raise awareness and help to better understand underlying causes and causalities regarding pain development, coping processes and corresponding pain behavior. Among common psychotherapeutic strategies, imaginative and hypnotherapeutic interventions appear to be encouraging [121]. The cognitive behavior therapy also proved to have some effect in combination with a pharmacological therapy concept in SCI [122], [123]. Its fundamental assumption is based on the idea that both pain behavior and cognitive appraisal do have a crucial impact on perception and experience of pain and might also be responsive to therapeutic approaches [124]. Further examples for promising psychotherapeutic approaches, which are yet lacking evidence in the field of SCI, are the positive psychotherapy, the mindfulness training, and the acceptance and commitment therapy (ACT) [125], [126], [127], [128], [129]. Also, relaxation techniques are already a key element in psychological treatment of chronic pain in general.

**RECOMMENDATION 10: TRANSCRANIAL DIRECT-CURRENT STIMULATION**

INDICATION FOR THE TREATMENT OF NOCICEPTIVE PAIN

Degree of recommendation and related specifics: **n/a**

Strength of consent: **n/a**

*INDICATION FOR THE TREATMENT OF NEUROPATHIC PAIN*

Degree of recommendation and related specifics: ↔

Strength of consent: **strong**

BASIC INFORMATION AND BODY OF EVIDENCE

Transcranial direct-current stimulation (tDCS) is capable of reducing neuropathic pain in SCI [130], [131], [132], [133]. A recent meta-analysis [116] indeed confirmed these findings on the basis of two RCTs [131], [132], which had a sufficient methodological quality. tDCS is well-tolerated, with instantly emerging mild headache of limited duration as the only described side effect of relevance. Notwithstanding the existing evidence, tDCS may only be considered as current third-line therapy. This is primarily explained by the availability of at least equally effective pharmacological treatment options, which are not only clinically well-established, but also well-tolerated. Furthermore, tDCS is not widely available, and experience in clinical routine is consequently meagre.

**RECOMMENDATION 11: TRANSCUTANEOUS ELECTRICAL NERVE STIMULATION**

INDICATION FOR THE TREATMENT OF NOCICEPTIVE PAIN

Degree of recommendation and related specifics: ↔

Strength of consent: **strong**

*INDICATION FOR THE TREATMENT OF NEUROPATHIC PAIN*

Degree of recommendation and related specifics: ↔

Strength of consent: **strong**

BASIC INFORMATION AND BODY OF EVIDENCE

The therapeutic value of transcutaneous electrical nerve stimulation (TENS) in therapy of pain after SCI is still being discussed. Even though certain positive effects on neuropathic pain were reported, the conclusiveness based on one RCT (n=33) [134], one prospective controlled trial (n=24) [135], and one case series (n=31) [136] is limited due to substantial methodological shortcomings [116]. Moreover, investigations on possible long-term effects are completely missing. Undesirable side effects seem to be mild, but still are described to comprise a potential deterioration of spasticity and pain symptoms as well.

With regard to nociceptive pain, two clinical studies suggest a modulation of spasticity by application of TENS in SCI [137], [138]. However, results regarding the impact of TENS on nociceptive pain in other underlying diseases are very heterogenous [139], [140], [141], [142]. Thus, the application of TENS may be considered for treatment of neuropathic pain and nociceptive pain on a case-by-case basis, if alternative treatment options are not available.

**RECOMMENDATION 12: ACUPUNCTURE**

INDICATION FOR THE TREATMENT OF NOCICEPTIVE PAIN

Degree of recommendation and related specifics: ↔

Strength of consent: **strong**

*INDICATION FOR THE TREATMENT OF NEUROPATHIC PAIN*

Degree of recommendation and related specifics: ↔

Strength of consent: **strong**

BASIC INFORMATION AND BODY OF EVIDENCE

Acupuncture is being discussed to have a therapeutic value for both nociceptive pain and neuropathic pain. This is based on two RCTs and one prospective controlled trial for nociceptive pain [143], [144], [145], as well as on one RCT [146] and one retrospective study with pre-post comparison [147] for neuropathic pain. However, sham-acupuncture has been shown to be equally effective in nociceptive pain [147], [148]. In the light of lacking superiority over a sham-treatment and also due to methodological weaknesses, the application of acupuncture may be waived in SCI-related pain [116]. 

**RECOMMENDATION 13: MASSAGE, HEAT THERAPY AND OSTEOPATHY**

INDICATION FOR THE TREATMENT OF NOCICEPTIVE PAIN

Degree of recommendation and related specifics: ↔

Strength of consent: **strong**

*INDICATION FOR THE TREATMENT OF NEUROPATHIC PAIN*

Degree of recommendation and related specifics: ↔

Strength of consent: **strong**

BASIC INFORMATION AND BODY OF EVIDENCE

There is no convincing evidence proving efficacy of massage, heat therapy or osteopathy in SCI-related pain due to methodological weaknesses, such as differentiation of pain types, no randomization and small sample sizes or single-blinding. Positive effects on SCI-related pain are discussed and the need of accurately designed RCT is being emphasized [63], [115], [145], [149], [150]. Considering both, positive experiences in other chronic pain conditions and the good tolerability of these measures, their application may be considered for pain after SCI.

**RECOMMENDATION 14: HYDROTHERAPY**

INDICATION FOR THE TREATMENT OF NOCICEPTIVE PAIN

Degree of recommendation and related specifics: ↔

Strength of consent: **strong**

*INDICATION FOR THE TREATMENT OF NEUROPATHIC PAIN*

Degree of recommendation and related specifics: ↔

Strength of consent: **strong**

BASIC INFORMATION AND BODY OF EVIDENCE

Secondary literature frequently describes positive effects of hydrotherapy on pain and psyche. The microgravity in water is assumed to be crucial for reducing the complained pain symptoms. This might be achieved by decompression of body regions that are vulnerable to pressure sores or of bones that are characterized by protuberances, but also by the facilitation to voluntarily/actively move paretic limbs in the water. Usual water temperatures of 32°C/89.5°F might additionally alleviate spasticity and also nociceptive pain as a secondary consequence [151]. Accordingly, hydrotherapy may be useful to treat nociceptive pain, but not neuropathic pain.

**RECOMMENDATION 15: VISUAL ILLUSION AND MOTOR IMAGERY**

INDICATION FOR THE TREATMENT OF NOCICEPTIVE PAIN

Degree of recommendation and related specifics: ↔

Strength of consent: **strong**

*INDICATION FOR THE TREATMENT OF NEUROPATHIC PAIN*

Degree of recommendation and related specifics: ↔

Strength of consent: **strong**

BASIC INFORMATION AND BODY OF EVIDENCE

Visual illusion techniques to treat neuropathic pain in SCI have been shown to provide general pain relief in one RCT without a relevant carry-over effect [132]. Two pre-post comparison studies yielded controversial results [152], [153], which does not allow to draw any firm conclusions.

**RECOMMENDATION 16: TRANSCRANIAL MAGNETIC STIMULATION**

INDICATION FOR THE TREATMENT OF NOCICEPTIVE PAIN

Degree of recommendation and related specifics: **n/a**

Strength of consent: **n/a**

*INDICATION FOR THE TREATMENT OF NEUROPATHIC PAIN*

Degree of recommendation and related specifics: ↔

Strength of consent: **strong**

BASIC INFORMATION AND BODY OF EVIDENCE

Based on a recent Cochrane study, transcranial magnetic stimulation (TMS) cannot be recommended for the use in central neuropathic pain (below-level) [116]. This meta-analysis investigating non-pharmacological interventions for chronic pain in people with spinal cord injury solely considered one single-blinded randomized crossover study with respect to TMS (n=13), which delivered negative results [154]. Only one single RCT (n=12) discusses a significant positive effect of TMS on central neuropathic pain relief, still lacking superiority with regard to the sham-stimulation [155]. Due to methodological weaknesses, this study was not considered for the aforementioned meta-analysis. According to the literature available, the data situation regarding TMS is unclear.

**RECOMMENDATION 17: THERAPIES WITH ADVERSE RISK-BENEFIT RATIO AND/OR OBSOLESCENCE**

BASIC INFORMATION AND BODY OF EVIDENCE

Epidural spinal cord stimulation, deep brain stimulation, motor cortex stimulation, selective rhizotomy including dorsal root entry zone lesioning and myelotomy cannot be recommended for treatment of pain related to SCI. This is based on an insufficient level of evidence and missing RCTs in combination with an adverse risk-benefit ratio, especially when considering the invasiveness of the mentioned measures. Nevertheless, selected reasonable indications might not be ruled out on a case-by-case basis.

## Discussion

This clinical practice guideline was developed to provide a structured and solid basis for clinical assessment and reliable source for therapy of pain related to SCI in German-speaking countries. Even though this guideline is consensus-based and not developed by means of a systematic review of existing evidence, the guideline panel made every effort to meet the required terms for achieving an appropriate and rigorous evaluation of relevant literature as comprehensive and accurate as possible. Thus, the primary criterion for rating each of the proposed recommendations was the appraisal of identified evidence and existing literature, respectively. However, in case of conflicting considerations, for instance among members of the guideline panel, the final grade of recommendation could potentially differ from those that would be expected according to Table 2 (Tab. 2) and Table 3 (Tab. 3). In this respect, influencing factors were aspects concerning practicability and suitability of therapeutic approaches and interventions in clinical routine, ethical and economic considerations, as well as the appreciation of risk-benefit ratios, especially with regard to potential side effects. Such considerations were then stated in the background text of the respective recommendation.

### Classification and diagnosis

With respect to the taxonomy of pain related to SCI, the ISCIP classification is recommended (see statement 1.1) since it is widely accepted and clearly specifies the different pain types in relation to SCI [28], [156]. For example, the distinction between at- and below-level neuropathic pain is of particular importance, since it is a unique characteristic of pain in SCI and considered to be caused by different underlying mechanisms. While below-level neuropathic pain (more than three levels below the NLI) presents as central neuropathic pain caused by a spinal cord lesion, at-level neuropathic pain (within three levels relating to the NLI) is frequently due to lesions of both nerve roots/peripheral nerves and spinal cord lesions, potentially resulting in a mixed pain presentation with peripheral and central neuropathic pain [12], [13].

From a clinical point of view, the distinction between neuropathic pain and nociceptive pain is particularly challenging at the level of injury, and, in case of sensory incomplete lesions, also below-level. This is due to the fact that perceived pain in these regions, irrespective of its (sub-)type, formally meets one of the diagnostic criteria for neuropathic pain, which is the presence of pain in areas of abnormal sensory function [10], [11]. When using the recommended ISCIPD (see statement 2.1), the distinction of both neuropathic pain and nociceptive pain requires caution in these zones.

Pain is a frequent complication directly or indirectly caused by SCI-related impairments of motor and sensory function. Common causes are muscular atrophy and also immobilization in bed, e.g. due to pressure sores. Changes with regard to biomechanics, a possibly inadequate sitting position in the wheelchair (see also statement 6.8), non-physiological gait patterns or transfer techniques are further examples that need to be considered and addressed when evaluating pain as a (secondary) complication of SCI (see also statement 3.1) [157]. Occasionally, such pain sources could also trigger other pain types or vice versa and finally result in a worsening of other complications in SCI (e.g. spasticity) [12], [93].

### Clinical examination

The clinical examination of a patient with nociceptive pain (musculoskeletal/visceral/other nociceptive pain) (see statements 3.1 and 6.4) needs to include the inspection, palpation, functional testing, evaluation of spasticity, contractures, myosclerosis, and the assessment of myofascial trigger points, but should also comprise a critical evaluation of symptoms relating to digestion, bowel and bladder management.

If evaluating peripheral and central neuropathic pain in SCI (see statement 3.2), a precise neurological examination is highly relevant (e.g. assessment of evoked versus spontaneous pain). In this respect, competing causes of aforementioned neuropathic pain characteristics (e.g. allodynia in fibromyalgia) ought to be kept in mind as well [158], [159], [160]. Even in complete SCI according to the International Standards for Neurological Classification of Spinal Cord Injury (ISNCSCI) [29], below-level neuropathic pain can occur. This is most likely due to the fact that a clinically complete SCI (ASIA Impairment Scale A) does not necessarily imply a complete transection of all efferent and afferent nerve fibers within the spinal cord. This occurrence could be reflected by the so-called “zones of partial preservation (ZPP)” in ISNCSCI and is frequently referred to as a “dyscomplete” lesion [161], [162]. In case neuropathic pain is emerging within the chronic phase of SCI, the examiner ought to consider secondary complications of SCI as causative (e.g. syringomyelia or carpal tunnel syndrome) [12].

Supplemental questionnaires and scales can be used to screen for neuropathic pain or to create a standardized and systematic basis for follow-up examinations and to evaluate the success of a previously initiated therapy (e.g. the numeric rating scale (NRS), the visual analogue scale (VAS), and the Likert-scale) (see statement 3.3). As opposed to the douleur neuropathique 4 questions (DN4), the spinal cord injury pain instrument (SCIPI), which was specifically developed for screening of SCI-related neuropathic pain, has recently been validated in German and is publicly available [163].

Not only somatic, but also psychological factors could influence the emergence and maintenance of pain (see statements 2.2 and 2.3). Hence, chronic pain could contribute to psychological stress and even trigger psychoreactive disorders (e.g. adaptive disorder or anxiety disorder). But strain or mental disorders could also enhance the perception of chronic pain in individuals with SCI [57].

All these mentioned challenges in terms of the clinical evaluation of nociceptive pain and neuropathic pain, in combination with possible psychological influences increase the probability of diagnostic uncertainty. If so, a multidisciplinary approach, assisted by physical therapists, occupational therapists, psychologists, but also relevant medical specialists, such as neurologists, psychiatrists, urologists, physiatrists, orthopedic surgeons, and specialists for internal medicine should be chosen.

### Medical diagnostics

Besides imaging (see statement 4.1), there are still other selected laboratory tests available to evaluate nociceptive pain. Most of those are at least partially controversial with respect to their psychometric properties. These tests comprise the ultrasonography of myofascial trigger points, the analysis of heart rate variability, as well as thermography for assessing the impact of the autonomic nervous system on the pain occurrence [164]. Instrumented gait and movement analysis, along with functional electromyography might additionally help to justify further targeted diagnostics and/or physical interventions, including the indication of medical aids. Finally, instrumented muscle function testing could provide information on aspects like the maximal strength, endurance, and fatigability. This again might give early hints on possibly emerging secondary complications (see also recommendation 8). However, such approaches still lack dissemination and expertise among SCI centers.

For more detailed neuropathic pain evaluation, parts of the QST (see statement 4.3) might also be helpful in detecting neuropathic pain in the early phase of SCI (see statement 5.3) [54].

### Prediction and prevention of pain in SCI

To effectively reduce the likeliness of chronic disease course for nociceptive pain (see statement 5.2), all involved therapists and also medical specialists are required to counteract muscular atrophies, tendon contractions and weakening of muscles with preserved voluntary function as early as possible. If some loss of function has already occurred, e.g. due to long-term immobilization, it is important to encourage and support the patients in terms of cautiously returning towards the pre-existing activities of daily living, still aiming at the best recovery possible, however without creating other complications. For example, extensive mobilization bears the risk of overstraining connective tissue, muscles and joints, which can elicit musculoskeletal nociceptive pain.

With regard to neuropathic pain and according to statement 5.3 it seems appropriate to continuously monitor acutely injured patients with signs of allodynia, which might allow for a timely and effective treatment of neuropathic pain and potentially diminish the likeliness of a chronic disease course.

### Expectations on treatment and associated considerations

To meet the content of statement 6.1, aims of an intended therapy should mostly be developed based on an interdisciplinary approach and patient-centered considerations. In most cases, a partial pain relief appears to be a rational endeavor. Patients under treatment should continuously be supervised to ensure both their compliance and the therapeutic success. Given the frequent recurrence and the chronic disease course as a consequence of SCI, practitioners should take care of a comprehensive and accurate documentation of all emerging pain aspects and causalities (e.g. ICD-10 codes), to guarantee an adequate and sufficient supply of medication, as well as any other means/therapies to treat SCI-related pain (see statement 6.3), and to avoid recourse claims from health insurance funds.

Once the decision was taken to additionally address emerged nociceptive pain symptoms with adjuvants according to the WHO pain ladder, potential synergistic effects on both nociceptive pain and neuropathic pain should be considered (see statements 6.5 and 6.10). In contrast, adverse effects and relevant interactions of certain pharmacological agents, as well as moderate effect sizes should also play a role in therapeutic deliberations (see statements 6.6 and 6.11). For example, the administration of opioids could severely deteriorate neurogenic bowel dysfunction and lacks sufficient evidence for treatment of myofascial pain (see statement 7 and recommendations 3.1 and 3.2). Potential restrictions regarding drug approval need to be considered (e.g. no approval for long-term administration of metamizole in Germany) (see statements 6.5, 6.7 and 6.11). In case of an off-label use of pharmacological and non-pharmacological therapeutics, patients need to be informed appropriately.

Besides pharmacotherapy, the supply with appropriate assistive devices is highly relevant in the context of identifying potential pain causalities (see statement 6.8). One of the most critical aspects is the accuracy of wheelchair fitting, which has to be re-evaluated on a regular basis over the years. Particularly in the acute phase, the appropriate wheelchair should not be finally adapted until the final stage of neurological and functional recovery following acute SCI has been reached.

### Pharmacological options for treatment of SCI-related pain

As already indicated by statement 6.2, SCI-specific evidence in terms of pain therapy is based on only a relatively small number of valuable, valid, and reliable clinical trials summarized in five meta-analyses [19], [20], [21], [22], [23]. In case of pharmacological treatment, such evidence is largely limited to the application of anticonvulsants and antidepressants (see recommendations 1.1 through 2.3). Thus, therapeutic approaches in clinical routine are frequently related to own subjective clinical experiences or experiences from other underlying diseases. This clinical practice guideline was dedicated to consequently providing a comprehensive guidance for a reproducible management of pain in a standardized manner, even if relevant evidence is vague or missing. In case this clinical practice guideline is not sufficient yet to address certain pain presentations in a given case, the therapeutic strategy could still be oriented towards the general recommendations for treatment of pain [165], [166]. The CPGs for diagnostics and treatment of chronic neuropathic pain [100], as well as the CPG about the long-term administration of opioids in non-malignant pain [167] are also of relevance in this context. Respecting the increasingly ageing patient population that is suffering from SCI [168], the PRISCUS list, specifying inappropriate medications in elderly [169], should also be considered.

Concerning the pharmacological therapy in general and with regard to the treatment of multimorbid elderly patients in particular, the therapeutic concept should always be based on a reasoned strategy. This includes a careful risk-benefit analysis regarding the expected effect size in relation to possible side effects. In case of polypharmacy, potential adverse interactions between different agents ought to be kept in mind. Furthermore, several drugs noted and recommended above can have a specific impact on spinal cord injured individuals, e.g. deterioration of an existing neurogenic bowel and bladder dysfunction or an increasing respiratory impairment in case of cervical injuries (Table 8 (Tab. 8)).

### Non-pharmacological measures for treatment of SCI-related pain

As compared to pharmacological treatments for SCI-related pain, the situation in terms of relevant literature is even worse in reference to non-pharmacological measures. These measures commonly represent complementary tools in SCI pain management. For instance, such approaches should seriously be considered in the case of prevailing nociceptive pain, which frequently results from disorders of the musculoskeletal/locomotor system. Among the non-pharmacological therapy approaches, “physical activity, exercise and physiotherapeutic measures” are the only ones that received an “ought to” and “should” recommendation in this CPG, each depending on the specific pain type (see recommendation 8). Despite the fact of only few existing evidences, this was mainly justified by two reasons:

1. according to the literature available, the beneficial impact of these measures on chronic pain is not ambiguous when applied properly, and
2. long-term clinical experience, entailing a broad and favorable acceptance for such measures in both pain medicine and the field of SCI. For achieving a sufficient application of those measures in terms of an effective complementary therapy, it might make sense to evaluate regional sports rehabilitation programs suitable for respective SCI conditions.

Given the huge clinical relevance of pain in general and musculoskeletal nociceptive pain in particular, spinal cord injury centers and even associated rehabilitation clinics might consider to take up the issue of pain management in separate and specialized training programs for their healthcare professionals. Such programs would enable the facilities to impart knowledge about the benign character, but also the pain-associated dangers and challenges of structural degenerations, which again might lead to a successive loss of function, activity, and finally participation.

### Summary of pharmacological and non-pharmacological treatments for neuropathic pain 

As illustrated by the results of the consensus building for this CPG, effective and lasting treatment options for neuropathic pain are limited. Table 9 (Tab. 9) summarizes the given agents and complementary measures for treatment of SCI-related neuropathic pain, arranged according to the degree of recommendation and divided into first- through to fourth-line therapies, as well as therapies that cannot be recommended.

## Conclusion

**Figure 2:**
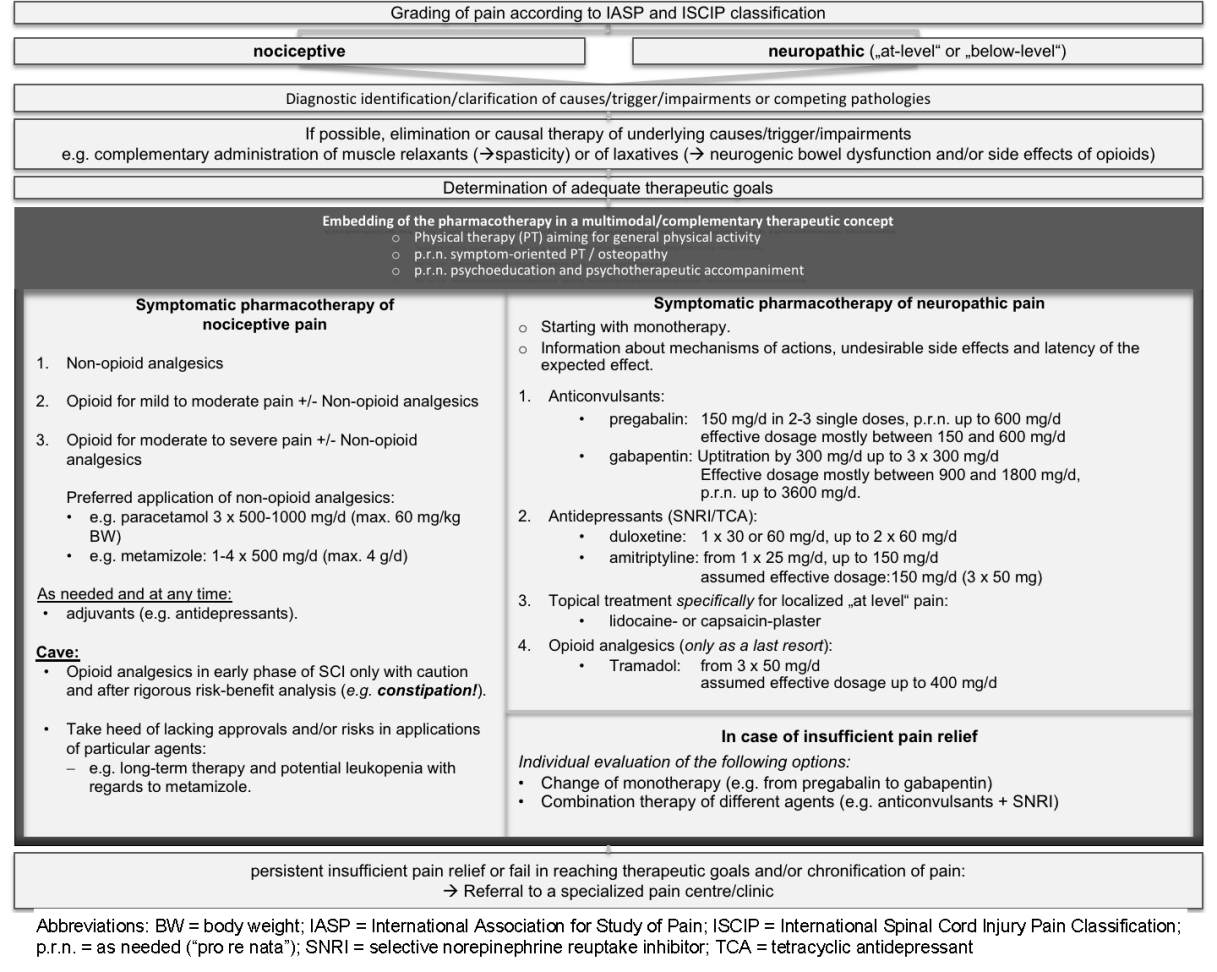
Clinical algorithm for assessment and management of individuals with pain after spinal cord injury

In summary, the overall evidence in respect to SCI-related pain is still sparse, which underlines the necessity for accurately designed RCTs investigating pharmacological and non-pharmacological treatment strategies. Meanwhile, a strictly developed clinical practice guideline may support two highly relevant endeavors:

1. It represents a clinical guidance for healthcare professionals in daily routine. This is illustrated by the clinical pathway in Figure 2 (Fig. 2), which is fundamentally based on a multimodal therapeutic approach.
2. It may serve as basis towards a gradual and ongoing improvement of pain management in SCI, as it reveals the most crucial gaps of evidence that ought to be primarily addressed in conceivable future trials.

## Notes

### Original (German) version

This guideline is also available for free download in German language on the AWMF website (register number 179/006): https://www.awmf.org/uploads/tx_szleitlinien/179-006l_S2k_Schmerzen_Querschnittlaehmung_2018-08.pdf.

### Funding

Financing was completely provided by resources of the DMGP. To preserve independence and neutrality, no representatives of the pharmaceutical industry were involved in the guideline development and consensus process, respectively.

### Competing interests

Potential conflicts of interest are listed in the guideline report published in German language on the AWMF website: https://www.awmf.org/uploads/tx_szleitlinien/179-006m_S2k_Schmerzen_Querschnittlaehmung_2018-08.pdf.

## Figures and Tables

**Table 1:**
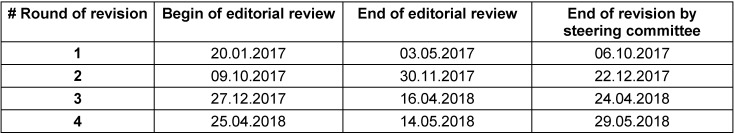
Review process within the clinical practice guideline panel

**Table 2:**
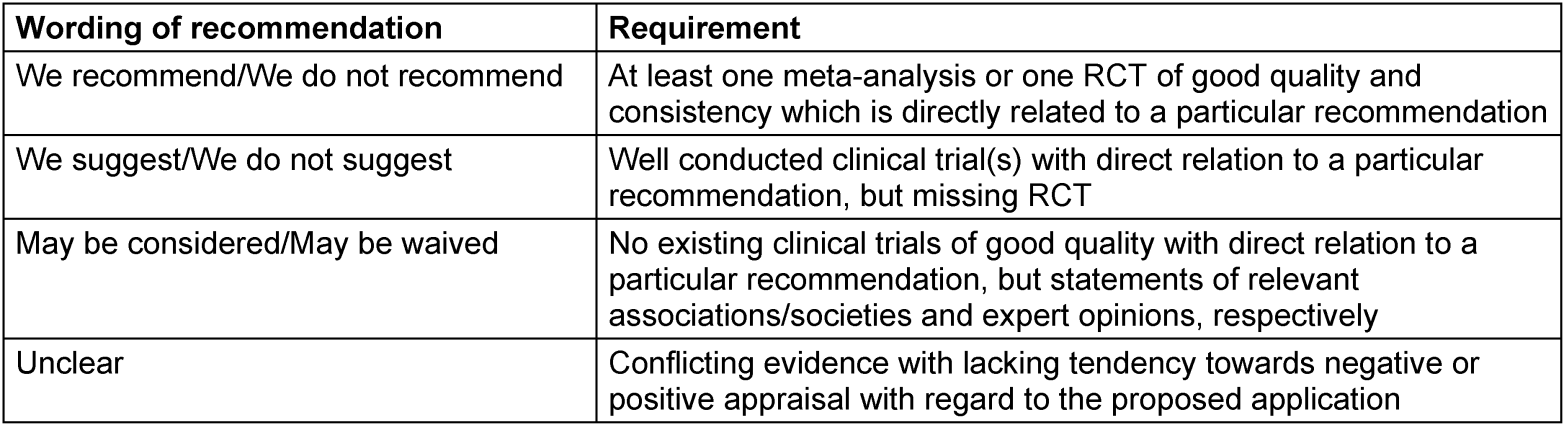
Guidance for the appraisal of recommendations

**Table 3:**
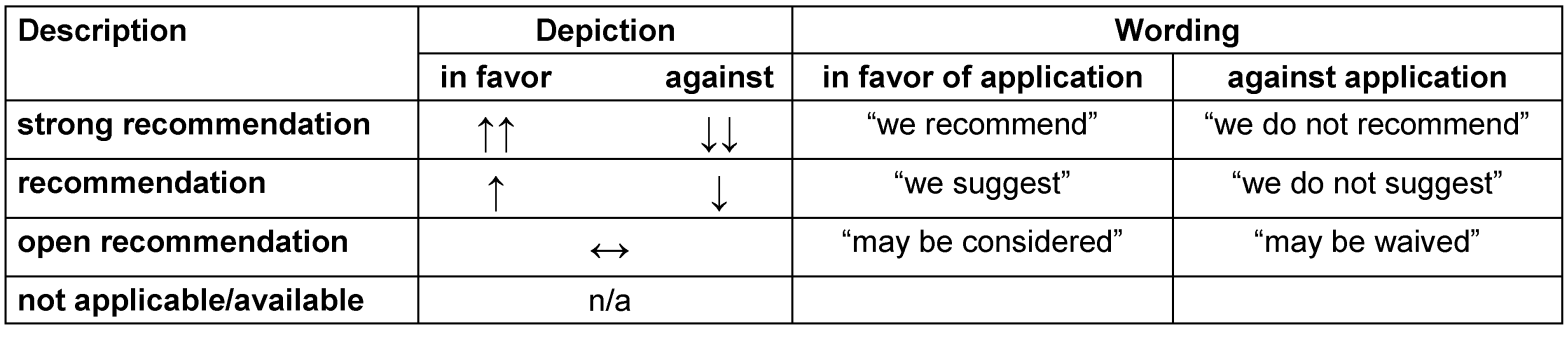
Grading of recommendations

**Table 4:**
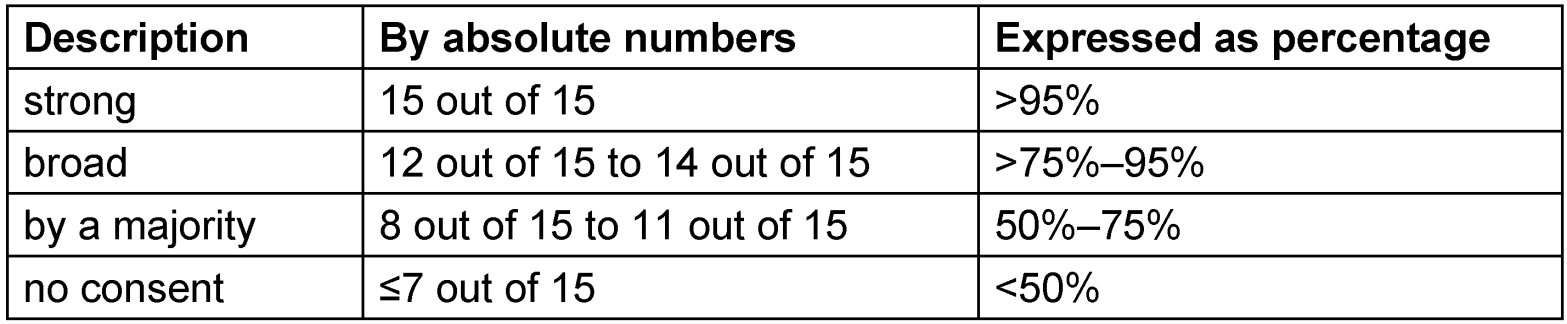
Rating of reached consent

**Table 5:**
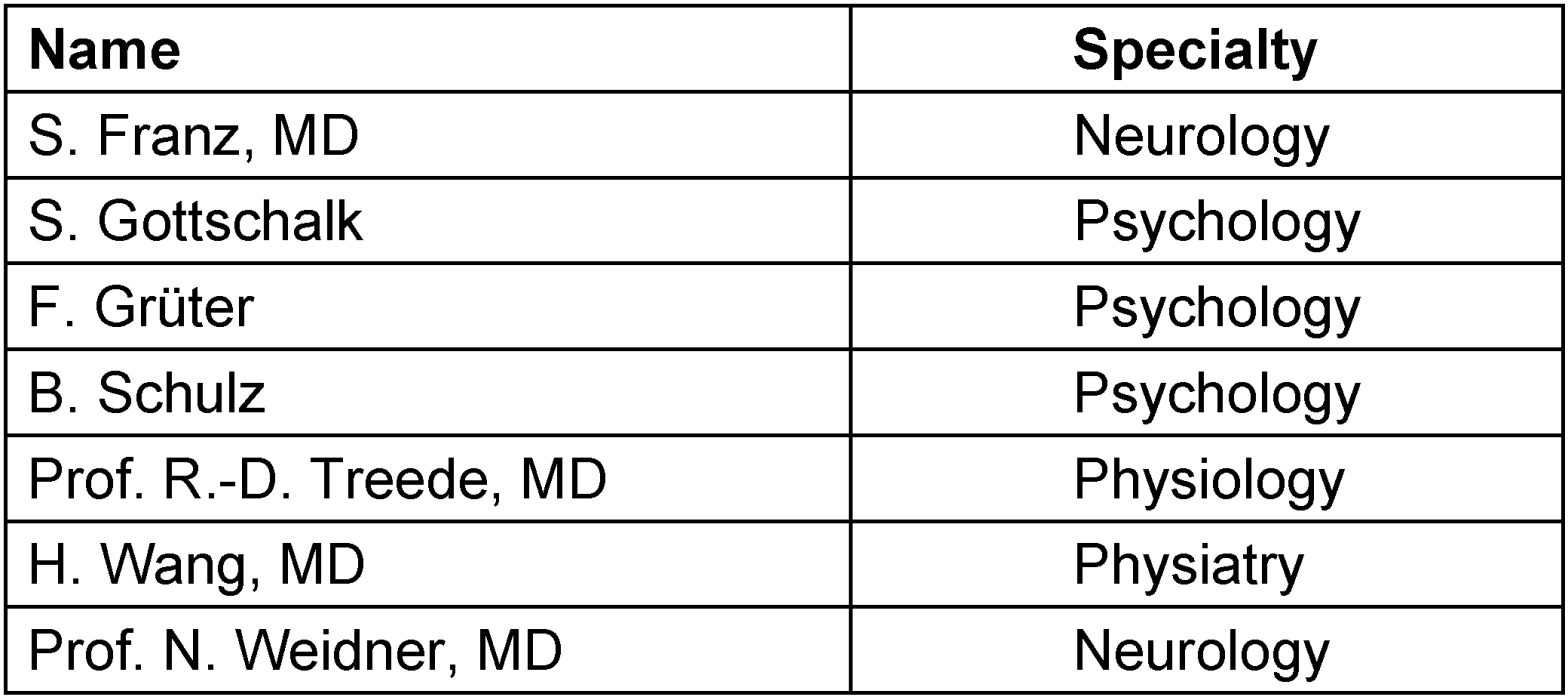
Members of the steering committee

**Table 6:**
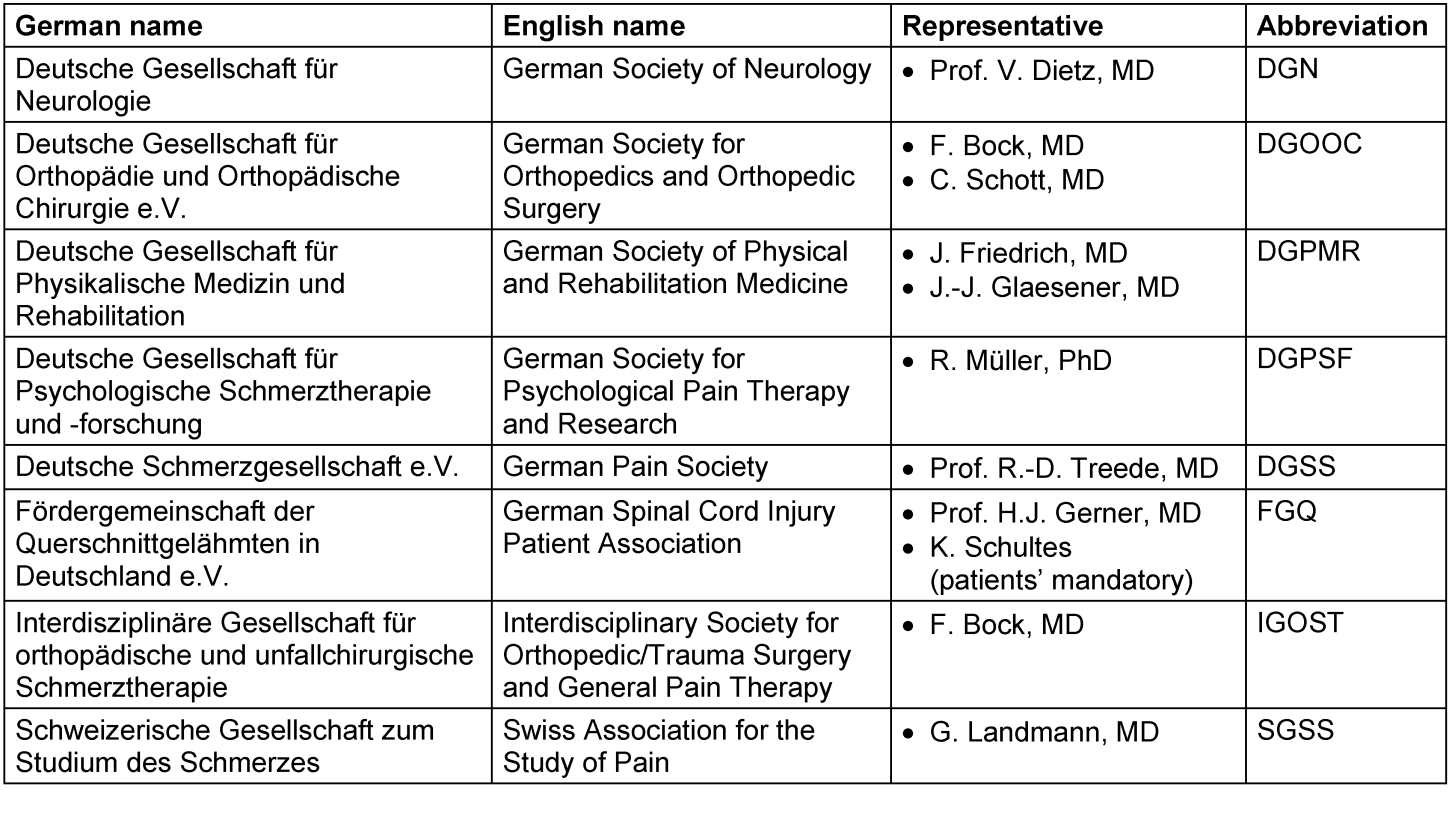
Listing of participating societies/organizations and their representatives

**Table 7:**
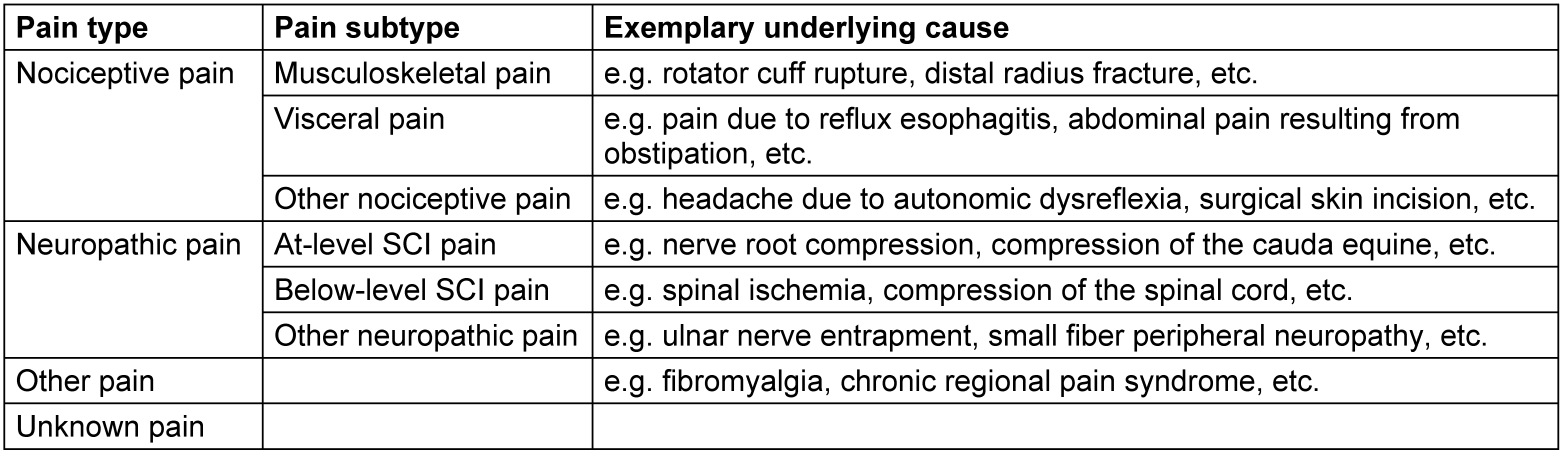
International Spinal Cord Injury Pain (ISCIP) Classification [30], [31]

**Table 8:**
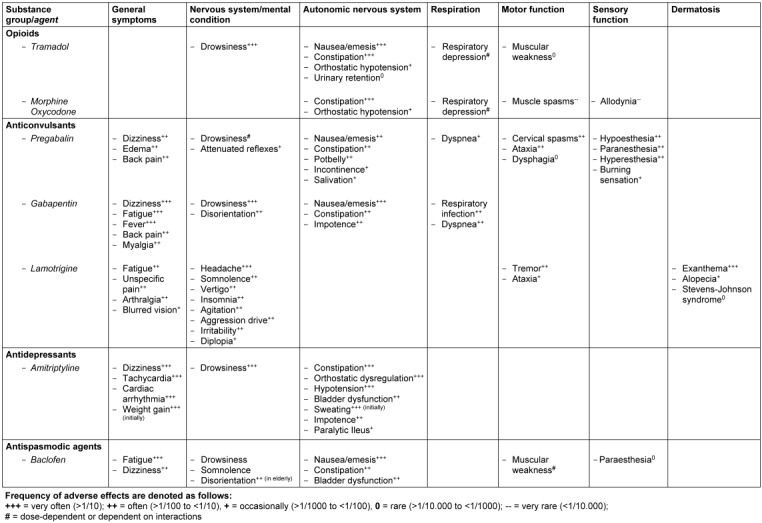
Substance-related undesirable side effects in treatment of neuropathic pain of particular relevance for SCI

**Table 9:**
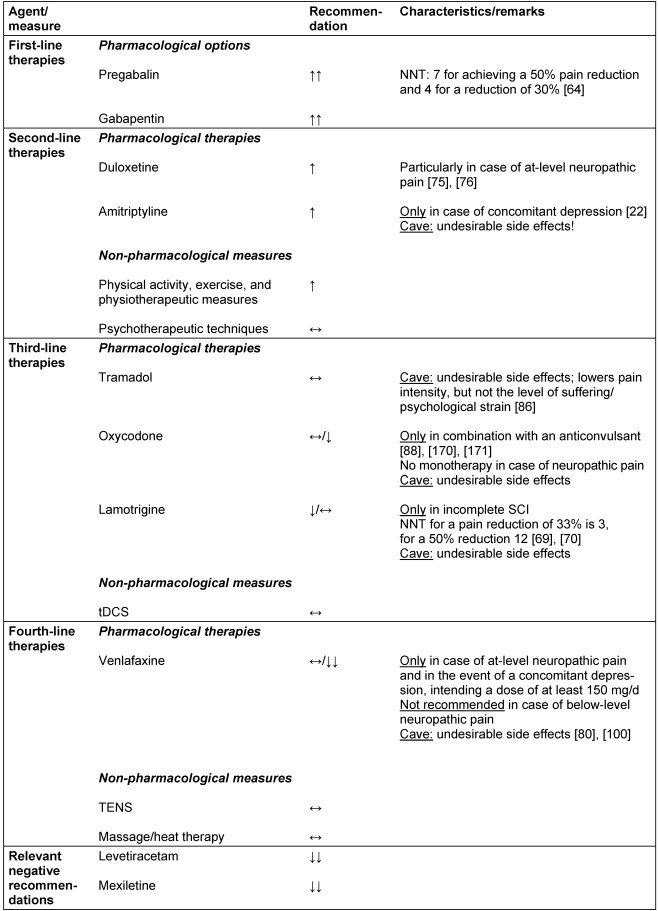
Recommendations for selected substances and measures in the therapy of SCI-related neuropathic pain
